# THE COSTS OF GENERATIVITY FOR OLDER ADULTS’ MENTAL HEALTH DURING THE COVID-19 PANDEMIC

**DOI:** 10.1093/geroni/igad104.0240

**Published:** 2023-12-21

**Authors:** Christine So, Katherine Fiori, Amy Rauer, Christina Marini

**Affiliations:** Adelphi University, Long Island City, New York, United States; Adelphi University, Garden City, New York, United States; University of Tennessee, Knoxville, Tennessee, United States; Adelphi University, Garden City, New York, United States

## Abstract

Generativity, the capacity to be productive, caring, and concerned with the well-being of the next generation, promotes positive mental health and posttraumatic growth in later life (Bellizzi, 2004). Generativity may be particularly important in recent years as older adults have faced the pandemic and its aftermath. For example, our previous work showed that generativity was associated with better mental health concurrently during this initial post-vaccine adjustment period (June - September 2021; So et al., 2022). The current study builds upon this work by using longitudinal evidence from a community sample of 95 older adults (M age = 68.4, range 50-91; 68% females; 93% White) to explore whether generativity predicted older adults’ anxiety, depressive symptoms, and life satisfaction six months later (December 2021-February 2022). Using structural equation modeling controlling for age, gender, and baseline depressive symptoms, we found that generativity positively predicted depressive symptoms six months later. In contrast, generativity at baseline was not associated with anxiety or life satisfaction six months later. It is possible that older adults who were more generative at baseline (when many still believed that COVID-19 pandemic would end with vaccinations) reported more depressive symptoms six months later when the highly contagious Omicron variant took over. Our findings thus underscore the complexity of the role of generativity in mental health outcomes, with associations sensitive to historical events.

